# Enzyme-Enhanced Electrochemical Immunoassay for Phenytoin

**DOI:** 10.6028/jres.093.159

**Published:** 1988-12-01

**Authors:** Mirtha Umaña, Jess Waller, Mansukh Wani, Carol Whisnant, Edgar Cook

**Affiliations:** Research Triangle Institute, Post Office Box 12194, Research Triangle Park, NC 27709-2194

## Introduction

The use of electrochemically generated electron-transfer mediators to control the oxidation states of enzymes has been the subject of considerable research [1,2]. Among these studies, the oxidation of glucose by glucose oxidase (GOx) has been extensively studied. Ferrocene derivatives [3] and other redox compounds [4,5] have been used to mediate the oxidation of dissolved and immobilized GOx [6]. Polypyrrole has been used to immobilize GOx on the surface of electrodes [7,8,9]. This polymer provides a simple and effective immobilization medium, and because it is an electronic conductor, it is expected to facilitate the electron transfer to the enzyme.

**Figure 1:**
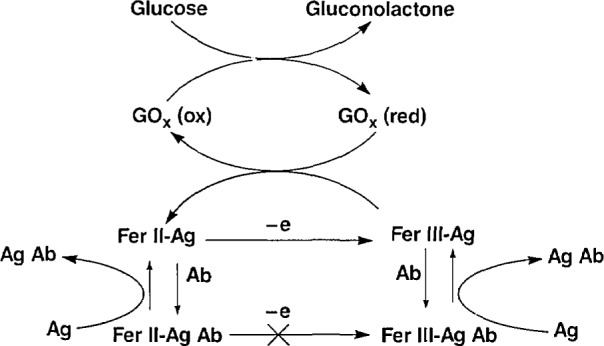
The catalytic cycle of GOx using ferrocene derivatives as electron-transfer mediators interrupted by the binding of an antibody to a ferrocene-antigen conjugate.

An important application of enzyme-mediated electrocatalysis is in immunoassays where the current amplification of the enzyme reaction enhances the sensitivity of the measurement. The immunological utility of the ferrocene/GOx system has been demonstrated with lidocaine as the antigen [10]. The general scheme is illustrated in figure 1; ferrocene-antigen (Fer-Ag) is competitively displaced from the antibody (Ab) by Ag, the analyte, and it is determined as an enzyme-amplified oxidation current. This scheme has significance because it expands the applicability of electrocatalytic analysis to analytes currently determined by immunoassays, and thus, deserves further research to demonstrate its generality.

This paper describes experiments aimed at understanding the kinetics and mechanism of the reaction, at determining the optimum conditions for the assay, and at testing the generality of the scheme shown in figure 1. We have chosen phenytoin [5,5-diphenylhydantoin (DPH)] as the model antigen, because of its clinical importance. For this drug to be effective and safe, plasma levels must be between 5 and 20 mg/L [11]. Thus, the development of easy, real-time assays for this and other critical-level drugs is highly desirable.

This paper also describes preliminary results on the electron-transfer mediation of ferrocene derivatives to polypyrrole-immobilized GOx. The goal of these experiments is to couple the polypyrrole-immobilized GOx to the ferrocene-DPH system to produce a reagentless electrochemical immunoassay sensor, so that easy, real-time determinations may be realized.

## Experimental

Electroanalysis was conducted with a PAR 175/173 potentiostat, in a three-electrode assembly (GC, Ag/AgCl, Pt). A two-compartment cell was used for immunological experiments. Preparation and use of GOx-polypyrrole modified electrodes (GOx/PP/GC) have been described elsewhere [7,8]. The polyclonal anti-DPH serum was prepared at RTI [12].

Ferrocene-phenytoin (Fer-DPH) was synthesized inhouse. To a room-temperature stirred solution of ferrocene carboxylic acid and 3-(2-aminoethyl)-5,5-diphenylhydantoin in anhydrous THF, 1,3-dicyclohexylcarbodiimide was added in one portion. After 5 h, the solvent was removed in vacuo. The residue was purified by elution from silica gel (5 g) using 20% acetone in hexane to yield a yellow solid with m.p. 248–251 °C. NMR, high resolution mass spectrometry, and elemental analysis confirmed the identity of the compound. Fer-DPH is insoluble in water but soluble in 10% CH_3_CN. Typical cyclic voltammograms (CVs) of DPH-Fer conjugate produced *E*°′ =0.47 V vs Ag/AgCl and 0.06 V peak separation.

## Results and Discussion

The DPH antibody binding was compared to that of Fer-DPH in a competitive inhibition RIA using ^3^H-DPH (specific activity 40.3 Ci/mmol/L) as tracer. Anti-DPH antibodies (DPH affinity > 10^9^ L/mol) showed 25% cross-reactivity (at 50% displacement of radioligand) with Fer-DPH. Thus, the Ab binding to Fer-DPH is sufficiently strong to form a stable Fer-DPH-Ab complex but DPH will readily displace Fer-DPH as needed for the scheme shown in figure 1.

The CVs of Fer-DPH are completely quenched by the formation of Fer-DPH-Ab upon addition of 100 *μ*L DPH antiserum. The inability of Fer-DPH-Ab to exchange electrons with the electrode may be due to steric effects, since the antibody may block the redox center from the electrode.

Kinetic information on the electrocatalysis was obtained with scan rate dependence studies. In the absence of GOx, the current (*i*_d_) is determined by diffusion of the ferrocene derivative and is expected to obey the Randles-Sevcik relationship for a reversible system:

$$i_{d}=2.69\times 10^{5}n^{3/2}AD^{1/2}C_{0}*\nu^{1/2},$$ (1)

where n is the number of electrons, *A* is the area of the electrode, *D* is the diffusion coefficient, *C*_0_* is the concentration of electroactive species in the solution, and *ν* is the scan rate.

In the presence of GOx and excess glucose, the limiting value of the current response (*i*_k_) is independent of *ν:*

$$i_{k}=n\;F\;A\;C_{0}*{\left(Dk^{\prime }C_{z}*\right)}^{1/2},$$ (2)

where *F* is the Faraday constant; *C*_z_* is the enzyme concentration, and *k*′ is the rate constant for the catalytic reaction. From scan rate dependence studies [13], we estimated a pseudo first-order rate constant for the catalytic reaction of GOx with ferrocene carboxylic acid and Fer-DPH of approximately 2 × 10^5^ and 4 × 10^5^ mol^−1^L^−1^, respectively. These values are comparable to the ones previously reported for ferrocene derivatives [5]. In the presence of 1.5 mmol/L GOx, the catalytic current is about three times the Fer-DPH background current and increases with enzyme concentration but is independent of glucose (above 20 mmol/L). Thus, the overall current is governed by the rate of reaction of the ferrocene with GOx rather than the rate of reaction of GOx with glucose as needed for the scheme of figure 1.

**Figure 2:**
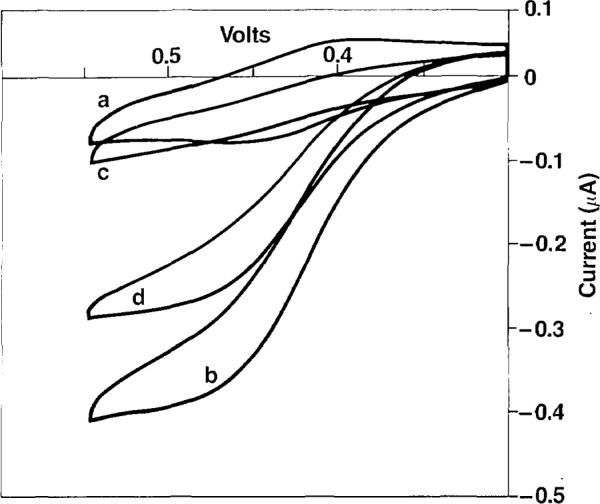
Cyclic voltammograms of Fer-DPH conjugate in aqueous 0.5 mol/L KC1 at pH 7 with 10% CH_3_CN. a) 200 mmol/L Fer-DPH, b) after addition of 40 mmol/L glucose and 0.8 rnmol/L GOx, c) after addition of anti-DPH serum, d) after addition of DPH.

**Figure 3:**
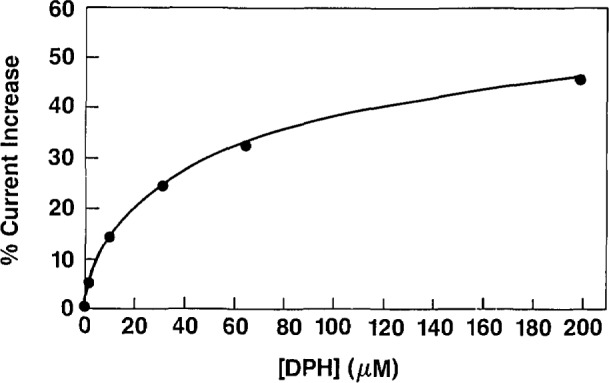
Calibration plot for electrochemical immunoassay of DPH.

Experiments shown in figure 2 demonstrate the development of the DPH electrochemical immunoassay. The CV of Fer-DPH is observed in curve a; upon addition of GOx and glucose, a large catalytic current is observed in curve b; upon addition of DPH-antiserum the catalytic current is almost completely inhibited in curve c; and after addition of DPH, due to the release of Fer-DPH from the antibody complex by the competitive binding of DPH, the catalytic current is recovered in curve d. The increase in current from curve c to curve d, is proportional to the amount of DPH added, and can be quantitated to produce a standard curve of DPH (pharmacologically relevant range) as shown in figure 3.

Currently, experiments aimed at producing a reagentless electrochemical immunoassay using a GOx/PP/GC electrode are in progress. GOx/PP/GC electrodes were examined for their response to ferrocene derivatives. The GOx/PP/GC electrodes were potentiostatted at 0.5 V, in an oxygen-free solution containing a measured amount of ferrocene. After addition of an excess of oxygen-free glucose solution, the current increased as expected from figure 1. The catalytic current increased linearly with ferrocene (l0^−4^ mol/L). Extrapolation to zero ferrocene shows a current increase (5 nA) due to the addition of glucose to a solution that contained no electron-transfer mediator. An explanation for this current increase may be that electrons are transferred directly from the polypyrrole that surrounds the GOx to its active center. This hypothesis is currently under investigation by monitoring the disappearance of glucose.
